# Editorial: Factors affecting graft survival after renal transplant: prevention of failure and follow-up strategies

**DOI:** 10.3389/fmed.2025.1569737

**Published:** 2025-02-21

**Authors:** Laila-Yasmin Mani

**Affiliations:** Department of Nephrology and Hypertension, Inselspital, Bern University Hospital, University of Bern, Bern, Switzerland

**Keywords:** kidney transplantation, IFTA, graft survival, viral infection, kidney donor, collapsing glomerulopathy, kidney MRI, arterial kinking

Since its first introduction in 1951 (1), kidney transplantation has become the best therapeutic option for patients affected by end-stage kidney disease. Indeed, kidney transplant recipients experience a clear survival benefit when compared to their matched counterparts on the waiting list (2). Thanks to the development of highly effective immunosuppressive regimens, the much-feared threat of acute organ rejection could be mastered. Surprisingly, however despite major advancements, a trend for decreased graft survival has been recorded over recent decades (3). Considered a final common pathway, the interstitial fibrosis and tubular atrophy (IFTA)-lesion of the kidney graft is thought to be multifactorial in origin secondary to immunological, cardiovascular, toxic and infectious causes (4). In this Research Topic, 15 articles of various formats from different geographic regions in the world invite us to shed light on diverse aspects of long-term kidney graft function.

Biopsy-proven causes for graft failure after a very long follow-up up to 26 years were examined by Betjes et al. in a prospective Dutch cohort of 737 kidney transplant recipients. The category of rejection accounted for the main part of death-censored graft failure while recipient's age, time after transplantation, and the presence of donor-specific antibodies before transplantation determined the relative contribution to overall graft loss and the type of rejection involved.

The influence of age and sex on graft survival was analyzed by Sancho et al. in a retrospective Spanish cohort of 1,101 kidney transplant recipients. The lower graft survival of female patients under 60 years of age was attributed to a more frequent use of expanded criteria donors and a higher prevalence of pre-transplant human leukocyte antigen sensitization.

Furthermore, the influence of donor race was examined in a retrospective clinico-pathological analysis from Columbia University NY on roughly 1,900 kidney transplant recipients. The authors confirmed a shorter allograft survival of kidney grafts from black donors and revealed a higher risk for the development of collapsing glomerulopathy in grafts from black donors (DiFranza et al.).

Infections as cause of late graft loss and complicated post-transplant course were the topic of several reports in this Research Topic. Brune et al. found no impact of 1^st^ year urinary tract infection (UTI) episodes with extended-spectrum beta-lactamase (ESBL) *Escherichia coli* and *Klebsiella species* on graft survival in 389 kidney transplant recipients within the Swiss Transplant Cohort while hospitalization and UTI recurrence rates were higher compared to patients affected by UTI with non-ESBL-producing strains. In an Italian retrospective cohort of 939 kidney transplant recipients, MRI-confirmed acute graft pyelonephritis was associated with reduced death-censored graft survival influenced by donor age, multifocal presentation, and abcedation as well as anti-thymocyte globulin induction (Tarragoni et al.).

In addition to bacterial infections, viral infections are known to affect graft survival. Dai et al. report a case of graft loss after acute blood group antibody-dependent rejection in an ABO-incompatible living donor kidney graft recipient triggered by prolonged parvovirus B19 infection. In another case report by Hosek et al., postrenal acute kidney graft dysfunction was caused by cytomegalovirus (CMV)-positive nephrogenic adenoma of the transplant ureter and a potential link between the rare entities of CMV ureteritis and nephrogenic adenoma of the transplant ureter is discussed. BK virus infection is known to affect kidney graft outcomes. In their retrospective study in kidney transplant recipients from donation after circulatory death donors, Liu et al. use a machine-learning approach to identify risk factors for the progression of BK viruria to BK viremia.

Additionally, metabolic factors may affect graft survival. In their narrative review, Tang et al. describe the importance, risk factors, and current treatment options for post-transplant anemia. Zeng et al. conducted a prospective cohort study in 600 kidney transplant recipients and meta-analysis to evaluate the role of vitamin D-levels as predictor of graft loss.

Finally, functional kidney graft ischemia has been evoked as cause for the development of IFTA. In our center, we have examined the hypothesis that grafts are less oxygenated during the sitting position due to kinking or bending of the iliacal vessels analogous to iliacal claudication described in professional cyclists. Using a multiparametric functional kidney MRI protocol including blood oxygen level-dependent (BOLD)-MRI, diffusion-MRI and arterial spin labeling-MRI during neutral and flexed hip position, the Bent Knee Study showed an acute impact of hip flexion on graft perfusion and oxygenation (Mani et al.).

Immune-dependent factors play a well-known role for kidney graft survival with rejection episodes contributing to early and late graft loss. Therefore monitoring of immunosuppression has a major role in preventing rejection and avoiding infectious and toxic complications. Reineke et al. correlated Torque teno virus load in 106 kidney transplant recipients undergoing indication biopsies to histological findings and conclude that Torque teno virus load may reflect changes in immunosuppressive therapy even after the 1^st^ year post-transplant. In a proof-of-principle study by Born et al. in 39 kidney transplant recipients, the feasibility of tacrolimus monitoring in hair samples has been studied in order to allow self-collection by patients and reduce the frequency of medical visits. Füessl et al. report on the potential benefit of the twice-daily use of extended-release tacrolimus in a kidney transplant recipient identified as fast metabolizer for tacrolimus leading to normalized trough levels and area under the concentration-time curve and improved graft function.

Lastly, the prediction of graft survival was studied by Hiramitsu et al. using a prediction model for the ideal perioperative estimated glomerular filtration rate (eGFR) in a cohort of 1,174 living-donor kidney transplant recipients. In this study, the predicted ideal eGFR/actual eGFR at 1, 2, and 3 weeks after transplantation was predictive for graft loss.

Taken together, ongoing research efforts continue defining and refining optimal post-transplant care for kidney transplant recipients with the ultimate goal and challenge to achieve improved long-term kidney graft survival.

## References

[1] Kuss R Teinturier J Milliez P. Some attempts at kidney transplantation in man. Mem Acad Chir. (1951) 77:755–64. 14874917

[2] Wolfe RA Ashby VB Milford EL Ojo AO Ettenger RE Agodoa LY. Comparison of mortality in all patients on dialysis, patients on dialysis awaiting transplantation, and recipients of a first cadaveric transplant. N Engl J Med. (1999) 341:1725–30. 10.1056/NEJM199912023412303 10580071

[3] Meier-Kriesche H-U Schold JD Srinivas TR Kaplan B. Lack of improvement in renal allograft survival despite a marked decrease in acute rejection rates over the most recent era. Am J Transpl. (2004) 4:378–83. 10.1111/j.1600-6143.2004.00332.x 14961990

[4] Pascual J Pérez-Sáez MJ Mir M Crespo M. Chronic renal allograft injury: early detection, accurate diagnosis and management. Transplant Rev. (2012) 26:280–90. 10.1016/j.trre.2012.07.002 22902496

